# Protocol for imaging and analyzing the synaptic coverage of astrocytes with super-resolution STED microscopy in mouse brain slices

**DOI:** 10.1016/j.xpro.2026.104906

**Published:** 2026-10-21

**Authors:** Lucy Waldron, Maïna Garnero, David Mazaud, Nathalie Rouach

**Affiliations:** 1 Neuroglial Interactions in Cerebral Physiology and Pathologies, Center for Interdisciplinary Research in Biology, Collège de France, CNRS, INSERM, PSL-NEURO, Université PSL, 75005 Paris, France; 2 Doctoral School SdV N°657, Université PSL, Paris, France; 3 Doctoral School N°158, Sorbonne Université, Paris, France

**Keywords:** Microscopy, Neuroscience, Special Issue

## Abstract

Super-resolution has allowed us to image areas previously inaccessible due to the limits of standard microscopy techniques. Here, we present a protocol to determine the astroglial coverage of synapses. We detail the steps to obtain and label mouse brain slices followed by imaging using 3D stimulated emission depletion (STED) microscopy and analysis using a custom-made Fiji plug-in, with imaging at a lateral resolution of 80 nm. This technique ascertains the interaction between astrocytes and the glial cell’s role in signaling and brain region function.

## Highlights

• Procedure for preparing and immunolabeling fixed mouse brain slices for STED imaging
• Guidance for 3D-STED image acquisition and deconvolution
• Uses super-resolution microscopy as well as a custom Fiji plug-in
• Instructions to assess the astrocyte coverage of synapses within mouse brain slices

## Abstract (editor highlights)

Publisher’s note: Undertaking any experimental protocol requires adherence to local institutional guidelines for laboratory safety and ethics.

## Graphical abstract

**Figure:**
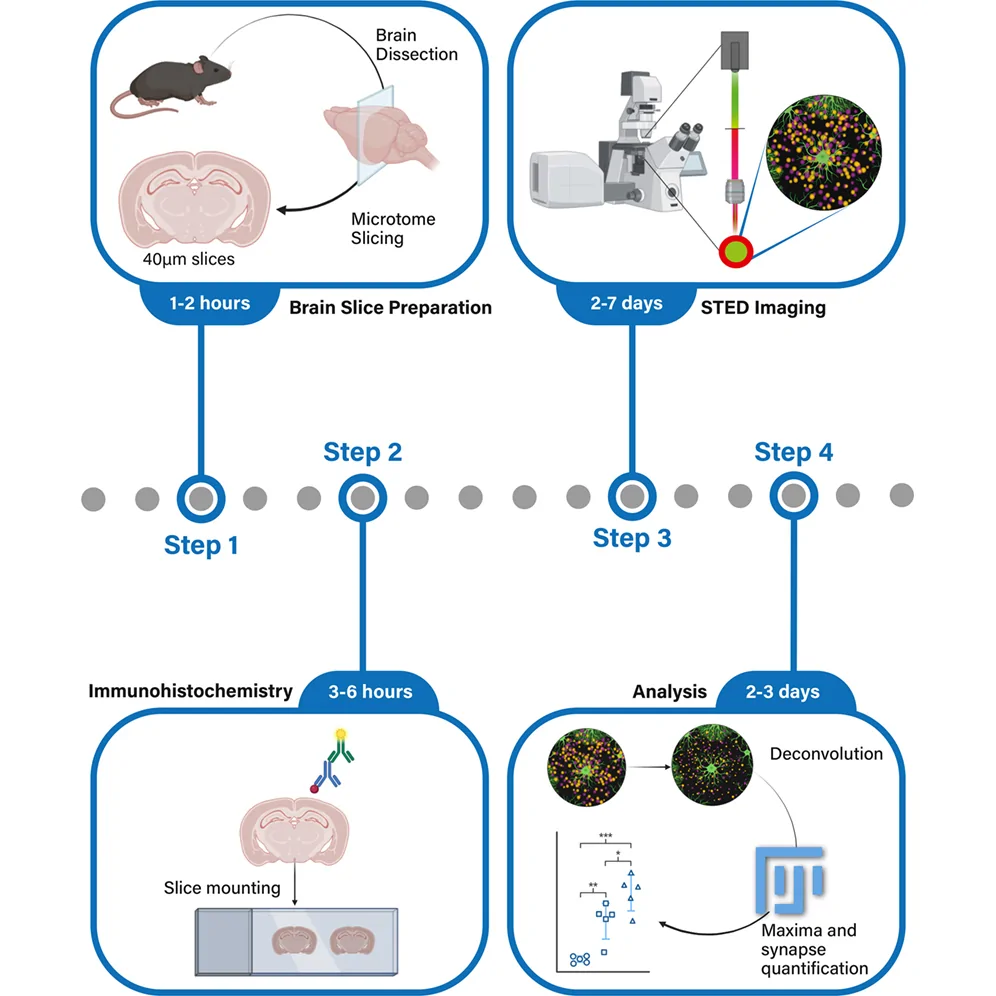

## Before you begin

This protocol describes step-by-step procedures to stain, image, and analyze astrocytic coverage of synapses in fixed mouse brain slices using 3D stimulated emission depletion (STED) super-resolution microscopy. This technique was developed using a GFAP-GFP transgenic mouse line^1^ but can be implemented in tissue with viral-mediated GFP-expression or in cell culture conditions with some modifications being made to the fixing or immunostaining protocols.

Astrocytes adopt a leaflet-like organization at sites of synaptic contact throughout their arborization, with fine astrocytic processes narrowing to approximately 200–250 nm in diameter.^2^^,^^3^ Here, 3D-STED microscopy (Figure 1A) is used because the fine peripheral and perisynaptic astrocytic processes that contact synapses are at or below the diffraction limit of conventional fluorescence microscopy.^3^^,^^4^ The improved lateral and axial resolution provided by 3D-STED enables more precise localization of astrocytic processes relative to pre and postsynaptic puncta, with our acquisition settings achieving 80 nm lateral resolution (Figure 1B). Although other super-resolution approaches may potentially be adapted to this workflow, the acquisition and analysis parameters described here were optimized for 3D-STED microscopy.

**Figure 1:**
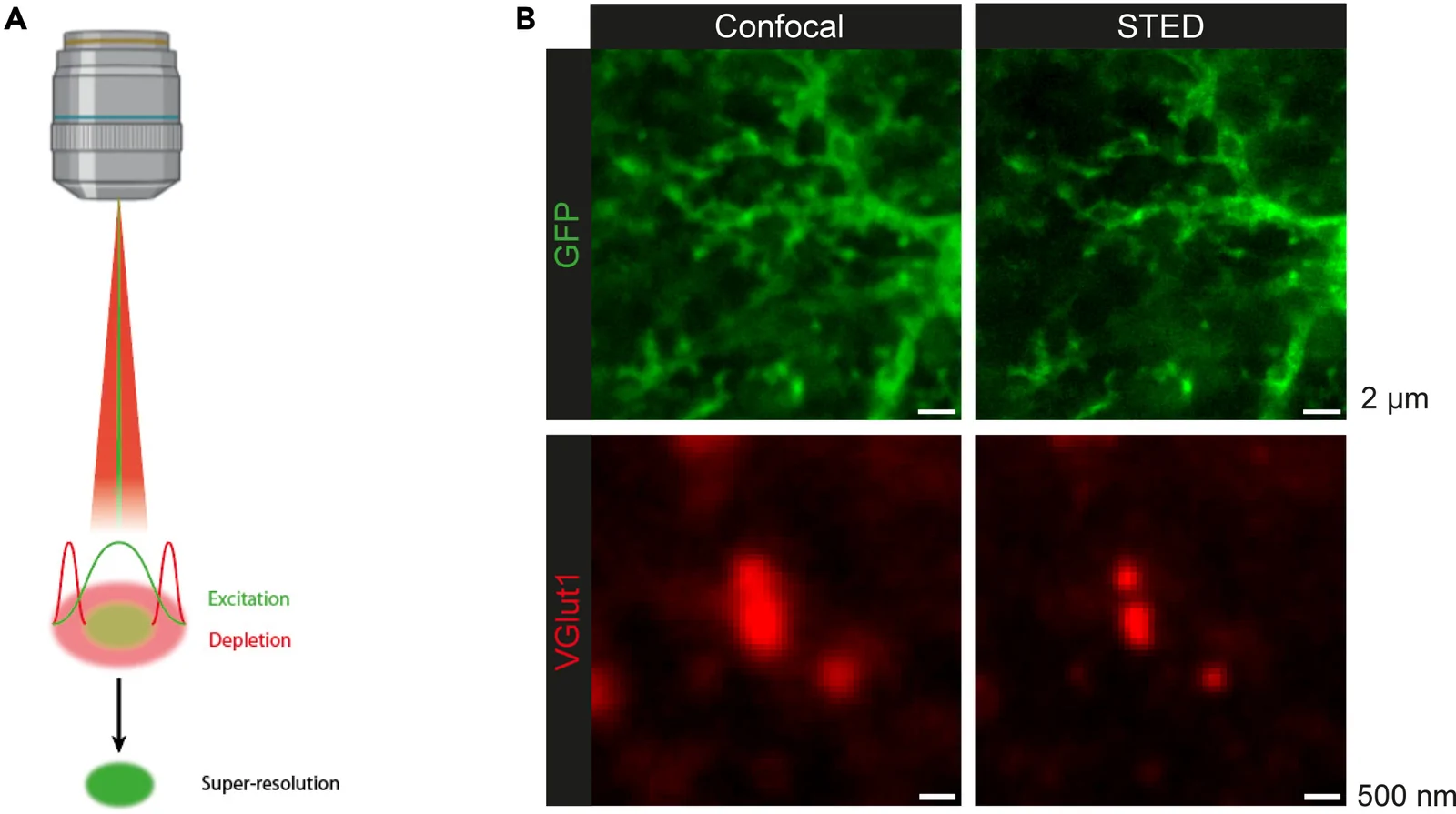
STED microscopy to study astrocytic coverage of synapses (A) Diagram of the principle of STED microscopy. (B) Examples of the increase in resolution in STED compared to confocal microscopy for astrocyte processes as well as VGlut1 dots. Scale bars: 2 µm (top), 500 nm (bottom).

This protocol was carried out on an Abberior Instrument super-resolution microscope (Expert Line) with their native Imspector software. The specific software commands and some hardware operations described below are therefore instrument-specific. Successful implementation on another microscope does not require an identical Abberior configuration but does require a system capable of 3D-STED imaging, with the same excitation (640 and 561 nm) and STED depletion (775 and 595 nm) lasers, a high-numerical-aperture objective compatible with the mounting medium, and acquisition of sufficiently sampled three-dimensional image stacks. Instrument-specific laser powers, detector settings, pixel dimensions, and acquisition parameters should be optimized accordingly.

Users familiar with conventional fluorescence microscopy should be able to follow the biological preparation and analysis portions of this protocol. However, STED acquisition requires appropriate instrument-specific training, particularly for optimization of excitation and depletion powers, sampling, alignment, and prevention of photobleaching. Initial microscope alignment and system calibration should be performed according to the manufacturer’s instructions or by appropriately trained facility personnel.

Because the aim of this protocol is to image individual astrocytes for which fine processes can be assigned unambiguously to a single cell, the labeling strategy should provide sufficiently sparse fluorescence. The GFAP-eGFP mouse line^1^ labels a subpopulation of astrocytes and may exhibit lower expression in certain brain regions (e.g., cortex), but typically provides sparser labeling suitable for single-cell imaging. When using viral approaches, sparse labeling can also be achieved by reducing viral titers or imaging on the edge of the injected area. Figure 3D provides examples of labeling suitable for ROI selection (right) and of denser labeling in which processes from neighboring astrocytes overlap (left). If sufficiently isolated astrocytes cannot be identified, alternative strategies are described in Troubleshooting 4.

### Innovation

This protocol provides an integrated workflow for quantifying the nanoscale spatial relationship between astrocytic processes and synapses in fixed mouse brain tissue. Although super-resolution microscopy has previously been used to visualize astrocytes and synaptic structures,^3^^,^^4^ the present protocol combines 3D-STED imaging of astrocytic, presynaptic, and postsynaptic markers, and automated image analysis within a single reproducible pipeline.

A key feature of the workflow is the simultaneous identification of putative synapses from the three-dimensional proximity of pre and postsynaptic puncta, followed by measurement of their distance to the segmented astrocytic surface. A custom Fiji plug-in automates puncta detection, confocal-based validation of STED detections, synapse assignment, astrocyte segmentation, and extraction of astrocyte-synapse distance measurements. This standardized workflow enables analysis of large numbers of synapses across multiple astrocytes and conditions.

The protocol also provides practical guidance for sparse astrocyte labeling, 3D-STED acquisition, deconvolution, parameter optimization, and statistical analysis. Together, these steps provide a transferable framework for investigating changes in synaptic coverage of astrocytes across brain regions, experimental conditions, and synaptic populations.

### Institutional permissions

Researchers utilizing this protocol must obtain approval from their institution’s Animal Ethics Committee. Experiments were carried out according to the guidelines of the European Community Council Directives of January 1st, 2013 (2010/63/EU) and approbation from the French Ministry of Research (APAFIS#19090-2019021211024807 v4). Efforts were made to minimize the number of used animals and their suffering. Experiments were performed in the supraoptic nucleus of GFAP-eGFP female mice, in which the enhanced green fluorescent protein (eGFP) is expressed under the GFAP promoter (provided by F. Kirchhoff, Max-Delbrück Center for Molecular Medicine, Berlin, Germany^1^). All mice had a C57BL/6J background and were group-housed on a 12-h light/dark cycle. Mice were perfused between 3–6 months.

### Perfusion

**Timing: 30 min preparation and 10 min per mouse**

1. Administer a lethal dose of sodium pentobarbital to the animal intraperitoneally.

**CRITICAL:** Sodium pentobarbital is a potent barbiturate and should only be handled by appropriately trained and authorized personnel and administered under a protocol approved by the relevant institutional Animal Ethics Committee. Wear appropriate personal protective equipment (PPE) and follow institutional regulations for its storage, use, record keeping, spill management, and disposal.

2. Transcardially perfuse with phosphate buffered saline (PBS) 1× (20 mL at 20°C–23°C) followed by 2% paraformaldehyde (PFA) in PBS 1× (30 mL at 20°C–23°C).

**CRITICAL:** PFA and formaldehyde-containing solutions are hazardous and should be handled using appropriate PPE and in an appropriate chemical fume hood when there is a risk of exposure to formaldehyde vapors. Consult the reagent safety data sheet (SDS) and institutional environmental health and safety guidelines for appropriate handling, spill procedures, and waste disposal. Materials and disposable items contaminated with PFA should also be disposed of according to institutional and local hazardous-waste procedures.

***Note:*** We use 2% PFA rather than 4% PFA to provide sufficient tissue fixation while limiting excessive crosslinking. In our experience, this milder fixation condition facilitates the preservation and visualization of astrocyte morphology, particularly fine astrocytic processes, including perisynaptic processes, which are critical for subsequent STED analysis.

3. Extract brain and postfix in 2% PFA for 12 h at 4°C.
4. Transfer tissue into 30% sucrose in PBS 1× and store at 4°C until fully equilibrated.

### Floating coronal sections

**Timing: 30 min per round**

5. Cut 40 μm thick brain coronal slices using a freezing microtome.

***Note:*** You can process several brains at once.

**CRITICAL:** Thin slices are needed for STED imaging. We use 40-μm-thick sections as a compromise between preservation of tissue integrity, efficient immunolabeling, and STED imaging quality. Section thicknesses of approximately 30–40 μm are expected to be compatible with this protocol. Thicker sections may also be suitable, but antibody penetration and STED imaging quality should be verified experimentally.

6. Store slices in PBS 1× + 1% sodium azide at 4°C (up to 6 months) or in a cryoprotectant medium (ethylene glycol 30%/glycerol 30 %/PBS 1×, see “materials and equipment” section) at −20°C (storage longer that 6 months).

**CRITICAL:** Sodium azide is hazardous and should be handled using appropriate PPE and in an appropriate chemical fume hood. Collect sodium-azide-containing solutions as hazardous chemical waste and follow the SDS and applicable institutional and local regulations for handling and disposal.

## Key resources table

**Table:** 

REAGENT or RESOURCE	SOURCE	IDENTIFIER
**Antibodies**		

Anti-vGlut1 (mouse) (dilution 1:200)	Synaptic Systems	CAT#135511; RRID: AB_887879
Anti-vGlut2 (mouse) (dilution 1:200)	Synaptic Systems	CAT#135421; RRID: AB_2619823
Anti-Homer1 (rabbit) (dilution 1:200)	Synaptic Systems	CAT#160003; RRID: AB_887730
Anti-Gephyrin (mouse) (dilution 1:200)	Synaptic Systems	CAT#147011; RRID: AB_887717
Anti-GAD (rabbit) (dilution 1:200)	Millipore	CAT#ABN904; RRID: AB_2893025
Anti-GFP (chicken) (dilution 1:200)	Aves Labs	CAT#1020; RRID: AB_10000240
Anti-rabbit Abberior STAR RED (dilution 1:200)	Abberior	CAT#STRED-1002-500UG; RRID: AB_2833015
Anti-mouse Abberior STAR RED (dilution 1:200)	Abberior	CAT#STRED-1001-500UG; RRID: AB_3068620
Anti-chicken Abberior STAR GREEN (dilution 1:200)	Abberior	CAT#STGREEN-1005-500UG
Anti-mouse F(ab’)_2_ Alexa Fluor 594 (dilution 1:200)	Interchim	CAT#715-586-150; RRID: AB_2340857
Anti-rabbit F(ab’)_2_ Alexa Fluor 594 (dilution 1:200)	Interchim	CAT#711-586-152; RRID: AB_2340622

**Chemicals, peptides, and recombinant proteins**		

Phosphate-buffered saline 10×	Euromedex	CAT#ET330
Paraformaldehyde	Euromedex	CAT#15714; CAS: 30525-89-4
Sodium azide	Sigma-Aldrich (Merck)	CAT#S8032-100MG; CAS: 26628-22-8
Euthasol Vet. (pentobarbital sodium)	Le Vet. B.V.	AMM: FR/V/9334132 5/2011
Mount solid antifade medium	Abberior	CAT#MM-2013-2X15ML
Saccharose	Sigma-Aldrich (Merck)	CAT#S0389; CAS: 57-50-1
Gelatin	VWR Chemicals	CAT#24350.262; CAS: 9000-70-8
Triton X-100	Sigma-Aldrich (Merck)	CAT#T9284; CAS: 9036-19-5
Ethylene glycol	Sigma-Aldrich (Merck)	CAT#324558-100ML; CAS: 107-21-1
Glycerol	Sigma-Aldrich (Merck)	CAT#G2025; CAS: 56-81-5
Ethanol 70%	Carlo Erba	CAT#528170; CAS: 64-17-5
Low Autofluorescence Immersion Oil, n = 1.518, Olympus Type F	Thorlabs	CAT#MOIL-30

**Experimental models: Organisms/strains**		

Mouse: *Mus musculus*, Tg(GFAP-EGFP)1Hket, 3-6 months, female	Pr. F. Kirchhoff^1^	MGI:6188855

**Software and algorithms**		

Imspector	Abberior Instruments GmbH	Imspector Image Acquisition & Analysis Software v16.3 https://imspector.abberior-instruments.com/; RRID: SCR_018863
Huygens	Scientific Volume Imaging	https://svi.nl/Huygens-Professional; RRID: SCR_014237
Fiji	Schindelin et al.^5^	https://doi.org/10.1038/nmeth.2019; RRID: SCR_002285
GraphPad	Graphpad	https://www.graphpad.com/; RRID: SCR_002798
3D Image Suite (MCIB3D)	Ollion et al.^6^	https://doi.org/10.1093/bioinformatics/btt276; RRID: SCR_024534
Fiji plugin Vglut_Homer_Astro3D v1.0.0	P. Mailly; this protocol	https://doi.org/10.5281/zenodo.21903313

**Other**		

Peristaltic pump	Masterflex	CAT#07555-05
Shaking platform Unimax 1010	Heidolph	CAT#10429692
Epredia Freezing microtome HM 450	Fisher Scientific	CAT#12072999
Star-Frost adhesive slides	Dutscher	CAT#100205
Corning Cover Glass	Corning	CAT#2980-244
595nm STED laser module	Abberior	N/A
775nm MPB-C pulse 40/80MHz	MPB-C	N/A
488nm, 561nm and 640nm excitation lasers (pulse 40/80MHz)	Abberior	N/A
Scientifica Microscope Body	Slice Scope Scientifica	N/A
Spatial Light Modulator – Easy3D	Abberior	N/A
Quad scanner	Abberior	N/A
Laser-Beamsplitter HC R785 1 lambda PV flat	AHF Analysetechnik	F38-786
CoolLED pE2 excitation system	Cool LED	N/A
Scientifica PatchStar micromanipulator	Scientifica	N/A
10 × Nikon CFI Plan Fluor NA 0.30 air objective	Nikon	MRH00105
100 × UPLXAPO100XO NA 1.40 oil immersion objective	Olympus	N5702400

## Materials and equipment

**Table:** Cryoprotectant solution

Reagent	Final concentration	Amount
Ethylene glycol > 99%	30% (v/v)	30 mL
Glycerol > 99%	30% (v/v)	30 mL
PBS 10×	1×	10 mL
ddH_2_O	N/A	30 mL
Total	N/A	100 mL

Store at −20C° for up to 2 years.

**Table:** Immunostaining buffer

Reagent	Final concentration	Amount
PBS 10×	1×	10 mL
Gelatin	0.002% (w/v)	0.2 g
Triton X-100 25%	0.25% (v/v)	1 mL
ddH_2_O	N/A	89 mL
Total	N/A	100 mL

Store at −4°C for up to 1 week.

## Step-by-step method details

### Immunohistochemistry

**Timing: 1–2 h, every day for 3 days**

This section describes the immunostaining of astrocytes and synaptic elements in fixed brain slices prior to STED imaging (Figure 2).

1. Incubate with primary antibodies.
  a. If slices were stored at 4°C in PBS 1× with 1% sodium azide, wash once in PBS 1× for 5 min at 20–23°C, with gentle agitation.
  b. If slices were stored in cryoprotectant medium, wash 3 times for 20 min in PBS 1× at 20–23°C, with gentle agitation.
  c. Incubate slices in immunostaining buffer (PBS 1× containing 0.002% gelatin and 0.25% Triton X-100, see “materials and equipment” section) for 1 h at 20–23°C, with gentle agitation, to permeabilize tissue and block non-specific binding sites.
  d. Incubate 12 h at 4°C, with gentle agitation, with primary antibodies diluted 1:200 in PBS 1× – gelatin 0.002% – Triton 0.25%. Incubate in a 24-well plate using 200 μL of antibody solution per well for up to three freely floating sections, ensuring that the sections remain fully immersed.
    i. Presynaptic element: Use anti-vGlut1 (Synaptic Systems, #135511, mouse) or anti-vGlut2 (Synaptic Systems, #135421, mouse) for excitatory synapses, or anti-GAD (Millipore, #ABN904, rabbit) for inhibitory synapses.
    ii. GFP amplification: Incubate with anti-GFP (Aves, #1020, chicken).
    iii. Postsynaptic element: Use anti-Homer1 (Synaptic Systems, #160003, rabbit) for excitatory synapses, or anti-Gephyrin (Synaptic Systems, #147011, mouse) for inhibitory synapses.

***Note:*** For labeling excitatory presynaptic elements, select either vGlut1 or vGlut2 based on the expression pattern in the brain region of interest.

2. Incubate with secondary antibodies.
  a. Wash slices once in PBS 1× for 5 min at 20–23°C, with gentle agitation.
  b. Wash 3 times for 10 min each in PBS 1× containing 0.002% gelatin and 0.25% Triton X-100 at 20–23°C, with gentle agitation.
  c. Incubate 12 h at 4°C, with gentle agitation, with secondary antibodies diluted 1:200 in PBS 1× – gelatin 0.002% – Triton 0.25%. Incubate in a 24-well plate using 200 μL of antibody solution per well for up to three freely floating sections, ensuring that the sections remain fully immersed.
    i. Presynaptic element: Use Abberior STAR RED goat anti-mouse (STRED-1001-500UG) for excitatory synapses, or Abberior STAR RED goat anti-rabbit (STRED-1002-500UG) for inhibitory synapses.
    ii. GFP amplification: Use Abberior STAR GREEN goat anti-chicken (STGREEN-1005-500UG).
    iii. Postsynaptic element: Use AF594 Donkey F(ab')_2_ anti-rabbit IgG (H+L) Min X (Interchim, #715-586-152) for excitatory synapses, or AF594 Donkey F(ab')_2_ anti-mouse IgG (H+L) Min X (Interchim, #715-586-150) for inhibitory synapses.

***Note:*** Here we use Abberior secondary antibodies as they are considered ideal for our Abberior microscope. However, any STED compatible secondaries may be used, such as Abberior STAR ORANGE.

**CRITICAL:** Accurate visualization of astrocyte morphology requires robust expression of a fluorescent reporter such as GFP. Signal intensity can be improved by post hoc amplification using anti-GFP immunostaining. Other green fluorescent proteins, including ZsGreen, mNeonGreen, or mStayGold, may also be suitable alternatives. ZsGreen can provide stronger signal than GFP in small astrocytic processes when expressed at optimized levels, however high expression levels should be avoided as ZsGreen tends to aggregate.

3. Mount the slices.
  a. Wash slices 3 times for 20 min each in PBS 1× at 20–23°C, with gentle agitation.
  b. Warm the Abberior Mount Solid Antifade (MM-2013-2X15ML) at 57°C for at least 15 min.
    ***Note:*** ProLong Gold can be used as an alternative mounting medium. When changing the mounting medium, STED image quality should be verified under the selected acquisition conditions.
  c. Place the brain sections directly onto StarFrost microscope slides. Add warmed Abberior Mount Solid Antifade and gently position a #1.5H Corning coverslip (24 × 40 mm; #2980-244) over the sections, avoiding air bubbles.
    ***Note:*** In this protocol, sections are mounted directly onto the microscope slide rather than directly onto the coverslip. This mounting configuration was used throughout our experiments and provided reliable STED imaging with 40-μm-thick sections. Direct mounting onto a #1.5H coverslip may reduce the optical path between the specimen and the objective. Therefore, changing the mounting configuration may require verification of STED image quality and acquisition settings.
  d. Seal the coverslip.
  e. Allow mounting medium to polymerize for 24–48 h in the dark at 20–23°C to obtain the appropriate refractive index.
  f. Store slides at 4°C in the dark until imaging.
    ***Note:*** This protocol has been successfully applied to different mouse brain regions and to astrocytes labeled either using the transgenic GFAP-eGFP reporter mouse line^1^ or viral approaches. It has also been successfully used with different lots of the antibodies listed above. The protocol has not been systematically validated in other species or with section thicknesses other than 40 μm. Changes in species, section thickness, reporter, antibody, or labeling strategy may affect antibody penetration, signal intensity, and background and may therefore require re-optimization of antibody concentration, permeabilization, and incubation conditions. Although the protocol has been successfully applied to different mouse brain regions, differences in target abundance between regions may also require adjustment of antibody concentrations.
    **CRITICAL:** Strong staining is required for STED microscopy due to high laser intensity. Therefore, GFP amplification steps, concentrated antibody solutions, and long incubations are critical. Antibody concentrations should first be optimized using relatively high primary and secondary antibody concentrations, typically 1/100–1/200. This initial step helps ensure dense and homogeneous labeling of the structure of interest. Antibody concentrations can then be progressively reduced to improve background and signal-to-noise ratio, but only after confirming that structural resolution is not compromised.
    **CRITICAL:** Always label the presynaptic element in the far-red channel. Far-red fluorophores (*e.g.*, Abberior STAR RED) are optimally depleted by the 775 nm STED laser and provide higher photostability and resolution. Assigning the presynaptic marker to this channel maximizes spatial precision for densely packed boutons.

**Figure 2:**
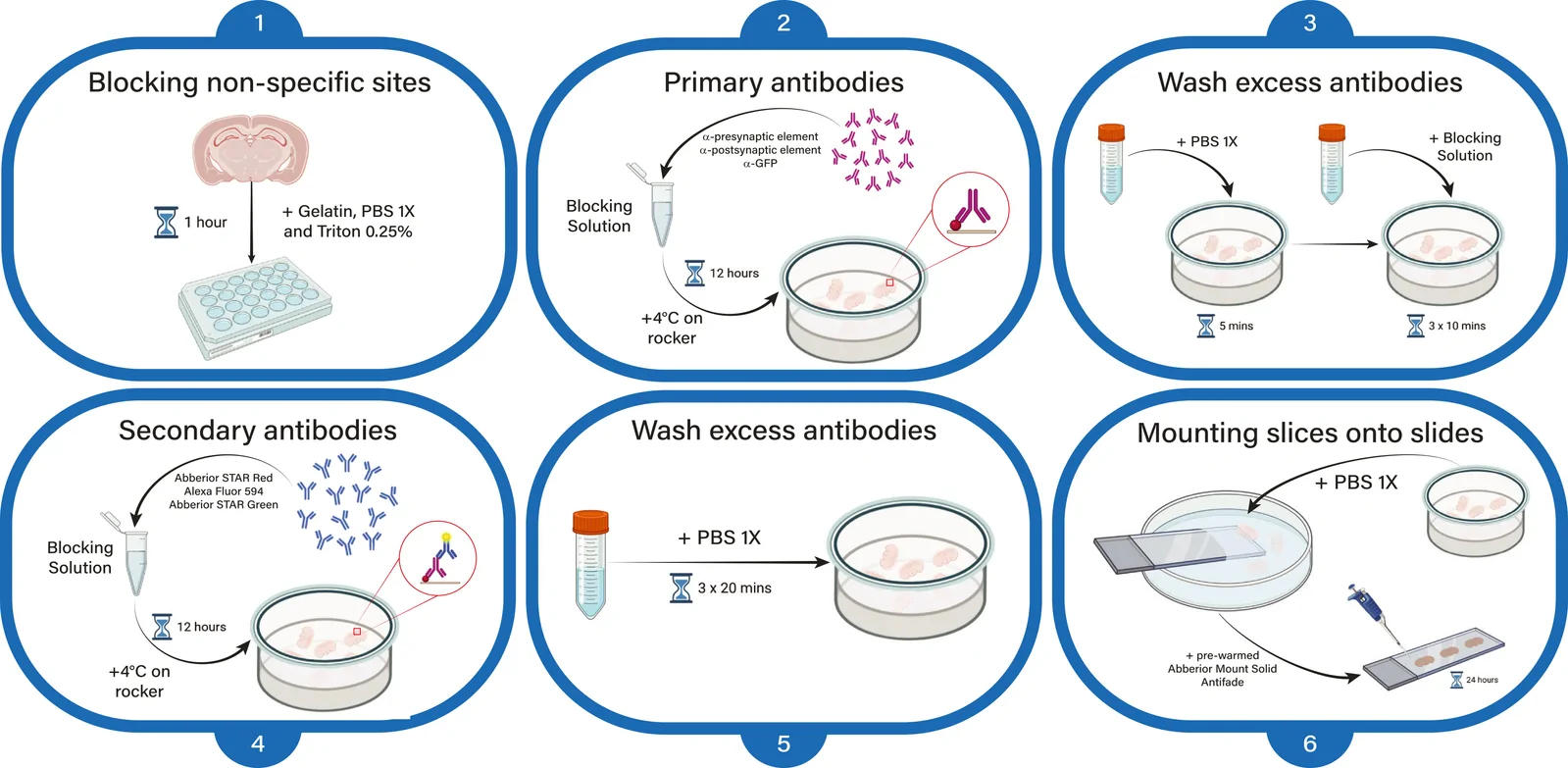
Overview of the immunostaining protocol

### Setting up the STED microscope

**Timing: 40 min**

This section describes microscope setup using our Abberior 3D-STED microscope and the version of Imspector software used in this protocol. The names and locations of software controls, as well as some hardware operations, are specific to this configuration and may differ on other STED systems. When adapting the protocol to another microscope, the key acquisition parameters to reproduce or optimize are the fluorophore/channel assignment, objective numerical aperture (NA), excitation and depletion powers, pixel size, z-step size, dwell time, imaging volume, and detector settings.

4. Turn on control cube and fluorescent lamp.
5. Turn on and set the LED of the CoolLED system to the 495nm wavelength on the control pod.

***Note:*** Navigating around the slice using the green channel is the easiest method unless you have also labelled with another structural indicator.

6. Launch the Imspector software (Abberior) and let the lasers warm up for approximately 30 minutes.
7. Open a new imaging window using the icon in the top left-hand corner of the window (Figure 3A).

**Figure 3:**
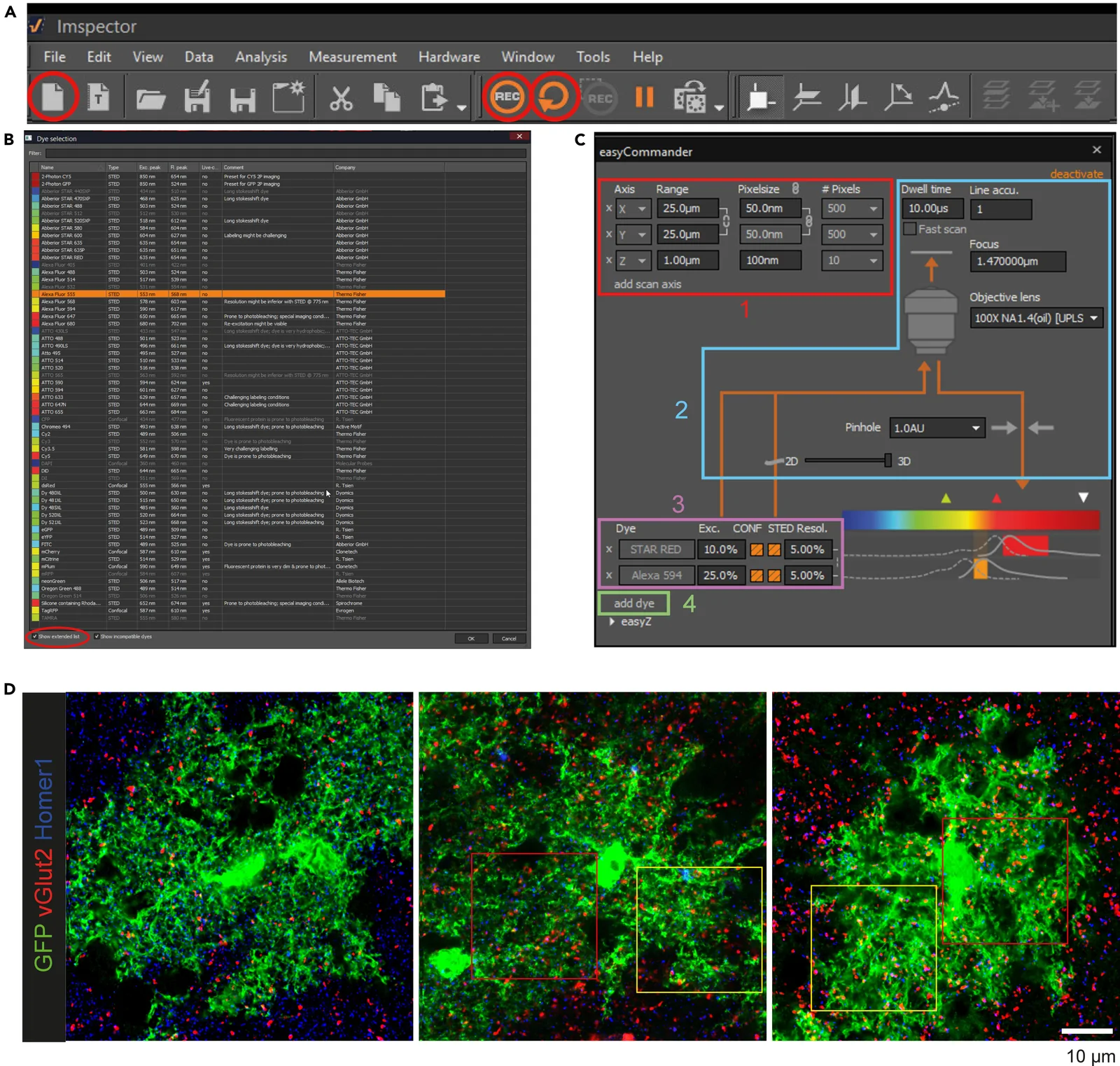
Setting up the microscope and imaging astrocytes with STED (A) Toolbar in Imspector software. Icons that are circled are, from left to right, open a new window, record a single image, repeating imaging. (B) Dye selection list with the extended dye list check box circled. (C) Image of the Easy Commander Live Dialog as well as the groups of parameters involved in this protocol. 1) Image Dimensions and Resolution. 2) Image acquisition parameters and objective specifications (this should be detected automatically). 3) Confocal/STED selection and laser power control. 4) Dye selection. (D) Representative examples of densely labeled astrocytes (left), moderately sparse labeling with partial overlap between neighboring astrocytes (middle), and an isolated astrocyte suitable for imaging (right). Yellow boxes indicate ROIs suitable for analysis and red boxes indicate ROIs that should be avoided. Scale bar: 10 µm.
8. Configure the acquisition using the Easy Commander Live dialog (Figure 3C).
  a. Click on the “Add Dye” button (Figure 3C, box 4) and select your dye from the list in the pop-up window (Figure 3B).
    ***Note:*** Click on “Show extended list” at the bottom left of this window, should your specific dye not be in the initial list.
  b. Click on “Add scan axis” (Figure 3C, box 1) to add a z-axis to STED acquisition.
  c. Set the image dimensions and scan axes (Figure 3C, box 1).
  d. Set the acquisition parameters (Figure 3C, box 2). The objective specifications should be detected automatically by the software.
    ***Note:*** Use full 3D-STED mode to improve resolution along the z-axis.
  e. Set the excitation and STED depletion laser powers for each channel (Figure 3C, box 3).
    ***Note:*** For your first image, set some basic settings, which should pick up some signal. Adjust to your particular tissue/cells on subsequent images.
9. Open the acquisition windows required for imaging according to the settings described in Table 1. In our configuration, we use three windows: one navigation window, one window containing the red and far-red channels, and one window for the green channel.

**Table 1:** Parameters for each window required for Vglut, Homer and Astrocyte imaging in confocal and STED

Setting	Navigation window	Far RED + RED channel		GREEN channel	ASTROCYTE image (OPTIONAL)		
Dye(s)	STAR GREEN	STAR RED	Alexa 594	STAR GREEN	STAR RED	Alexa 594	STAR GREEN
Laser Power (%)	5	10	20	10	5	15	5
Microscope Type(s)	Confocal	Confocal + STED		(Confocal) + STED	Confocal		
STED laser Power (%)	N/A	5		5	N/A		
Image size (xy μm)	80 × 80	25 × 25		25 × 25	80 × 80		
Pixel size (xy μm)	200	50		50	200		
Image size (z μm)	N/A	1		1	N/A		
3D STED (5%)	N/A	100		100	N/A		
Pixel size (z μm)	N/A	100		100	N/A		
Dwell time (μs)	10	10		10	10		
Line accumulations	1	1		1	1		
Pinhole size (AU)	1	1		0.8	1

***Note:*** A 4^th^ window can be used to image the whole astrocyte if needed.

***Note:*** Cut down on acquisition time by removing the confocal image from the green channel. It is not needed in further analysis.

***Note:*** Adjust excitation and depletion laser powers, dwell time and line accumulation based on the quality of the staining, signal-to-noise ratio, and susceptibility to photobleaching. If substantial photobleaching occurs during acquisition, see troubleshooting 5.

***Note:*** Adjust pixel sizes based on the on the magnification and the NA of your objective, respecting at least the Nyquist factor for sampling your structure.

### Imaging astrocytes with STED

**Timing: 2 days (for 4 slices with 5 astrocytes each), variable**

This section describes the imaging of astrocytes and synaptic elements using the Abberior 3D-STED microscope (see Table 2 for microscope setting details).

10. Screw in the 10× objective into the microscope stage.
11. Move the shutter lever to the fully “in” position.
12. Set the filter wheel to 2 to switch the microscope to ocular mode.
13. Place the slide within the microscope stage and secure with magnets.
14. Open the shutter.
15. Orient the slice and find the area of interest using the control cube.
16. Switch to the objective with the largest magnification.

***Note:*** A high NA 100× oil objective is ideal with corresponding mounting media.

17. Add oil or water to the slide (depending on your objective of choice).

***Note:*** Switch the control cube to a slower speed to focus and move around.

18. Find an isolated astrocyte within your zone of interest.

**CRITICAL:** The aim of this protocol is to image isolated astrocytes for which fine processes can be assigned unambiguously to a single cell (Figure 3D, right). Whenever possible, avoid cells with extensive overlap between processes from neighboring astrocytes (Figure 3D, left). If well-isolated astrocytes cannot be identified, alternative strategies are described in Troubleshooting 4.

19. Switch off the shutter.
20. Switch the microscope to detector mode by pulling the shutter level to the fully “out” position and turning the filter wheel to 1.
21. Click on your navigation window and press the live button.
22. Use the control cube to centre your astrocyte for imaging.
23. Find a place on the Z axis where the most astrocyte processes are seen.

***Note:*** This will usually be in the central plane of the astrocyte but may vary.

***Note:*** Use this plane as the center of the z-stack. We typically acquire a total z-range of 1 μm, extending 0.5 μm above and 0.5 μm below the selected plane, using a 100-nm z-step.

***Note:*** At this point you can take an overview of a single plane of your entire astrocyte for illustration or presentation purposes using your “Astrocyte image” window. We typically acquire a 70 × 70 μm overview image (Figure 3D).

24. Click on the live button once again to stop the recording.
25. Click and drag on your navigation window to define a rectangular region of interest (ROI).
26. Position the ROI within the peripheral arborization of the astrocyte.

***Note:*** We typically use ROIs of 25 × 25 μm in x and y, and 1 μm in z, with a 100-nm z-step.

**CRITICAL:** Astrocyte-synapse interactions occur throughout the astrocytic territory, with fine peripheral processes representing a particularly relevant compartment for assessing synaptic coverage, with a higher density of synaptic contacts.^7^ We therefore typically position ROIs toward the peripheral, fine-process-rich portions of the astrocyte (Figure 3D, right, yellow ROI), rather than over the soma and major proximal processes (Figure 3D, right, red ROI).

***Note:*** If completely isolated astrocytes are difficult to identify, cells with limited overlap may be used provided that the processes within the selected ROI can be unambiguously assigned to a single astrocyte. Select regions without visible overlap (Figure 3D, middle, yellow ROI) and avoid areas in which processes from neighboring astrocytes cannot be distinguished (Figure 3D, middle, red ROI).

27. Hover your cursor over the box and press “c” on your keyboard.
28. Click and drag your cursor over to the Far Red/Red channel window and release.

***Note:*** This method centers your images onto the center of the selected ROI. You retain the desired dimensions while modifying only the overall location of each image.

***Note:*** An icon should appear in place of your cursor. It should change from white to black when over the desired window.

29. Repeat step 28, but drag the cursor to the green window.
30. Click on your Far Red/Red channel window and press the “Rec” button.

***Note:*** All STED z-stacks used for three-dimensional astrocyte-synapse distance measurements are acquired in 3D-STED mode, providing super-resolution information in both the lateral and axial dimensions.

31. Once this is complete, click on the green channel window and press the “Rec” button.
32. Save both images.

***Note:*** The software will record over a previous image so make sure to save between ROIs/astrocytes.

***Note:*** Save the images as either a.tif or in the native.msr format.

***Note:*** Make sure to have a consistent naming convention to ease later analysis. *E.g*.: Condition_Animal Nbr_Slice Nbr_Astrocyte Nbr_ROI Nbr_FR-R.

33. Choose another ROI on a separate section of the astrocyte and repeat steps 25–32.
34. Repeat once more on a third section of the astrocyte.

***Note:*** Properly separate the ROIs to avoid bleaching from one ROI appearing on another.

35. Acquire images from three ROIs per astrocyte, with a minimum of four astrocytes per animal and at least four animals per experimental group.

***Note:*** The use of three ROIs per astrocyte was empirically selected to balance representative sampling of the astrocytic arborization with acquisition time and potential photobleaching. If the number or dimensions of ROIs are changed, they should be defined consistently across experimental groups.

36. Remove the slide and clean it with 70% ethanol.
37. Remove the objective and clean it with a lens tissue and a small amount of ethanol 70%.
38. Make sure all images are saved before closing down the software.
39. Turn off the fluorescent lamp.
40. Turn off the control cube.

**Table 2:** The different microscope parameters used in this protocol

Microscope settings	Far RED confocal	Far RED STED	RED confocal	RED STED	GREEN STED
X (nm)	50	50	50	50	50
Y (nm)	50	50	50	50	50
Z(nm)	100	100	100	100	100
Numerical Aperture ∗	1.4	1.4	1.4	1.4	1.4
Lens refractive index ∗	1.515	1.515	1.515	1.515	1.515
Medium refractive index	1.515	1.515	1.515	1.515	1.515
Objective Quality	Good	Good	Good	Good	Good
Coverslip position (μm)	−2.000	−2.000	−2.000	−2.000	−2.000
Imaging direction ∗	Upward	Upward	Upward	Upward	Upward
Microscope Type	Confocal	STED	Confocal	STED	STED
Multi-photon excitation	N	N	N	N	N
Back projected Pinhole radius ∗	280	280	280	280	280
Excitation wavelength	640	640	561	561	488
Emission wavelength	662	662	617	617	525
Excitation Fill factor ∗	2	1.2	2	1.2	1.2
STED Depletion mode	N/A	Pulsed	N/A	Pulsed	Pulsed
STED Saturation factor ∗	N/A	35	N/A	35	35
STED wavelength	N/A	775	N/A	775	595
STED immunity fraction ∗	N/A	8	N/A	8	8
STED 3×	N/A	100	N/A	100	100

Settings with asterisks are values which will need to be changed according to the parameters of your microscope/the objective used.

### Image processing

**Timing: 1 day**

### Deconvolution

**Timing: 2 h (approx. 20 astrocytes)**

41. Load up the Huygens Pro software.
42. Open the workflow processor (see Figure 4A).

**Figure 4:**
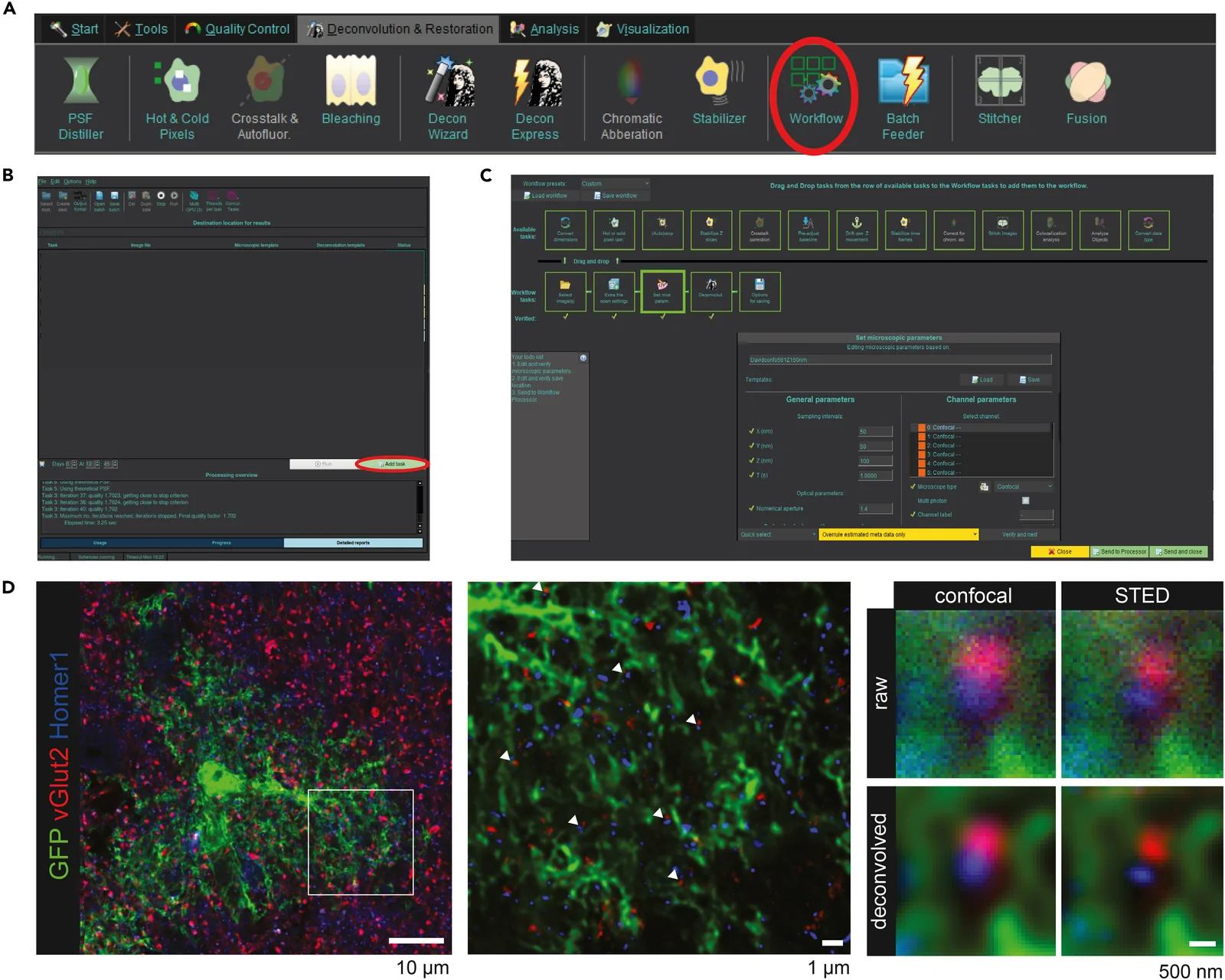
Deconvolution using Huygens Pro (A) Huygens Pro Toolbar with the workflow button circled. (B) Workflow processor home window. “Add task” button is circled. (C) Workflow Designer window. This shows all the different tabs which are essential to add to the workflow. (D) Representative examples of results obtained following deconvolution. Putative synapses are labelled with white arrows. Scale bars: 10 µm (left), 1 µm (middle), 500 nm (right).
43. Click on Add workflow (see Figure 4B).
44. Drag and drop these actions from the available tasks section in this order (see Figure 4C).
  a. Select images.
  b. Extra file open settings.
  c. Set microscope parameters.
  d. Deconvolution.
  e. Set save location.

***Note:*** You can add other parameters such as drift Z correction but these are the basic essentials for deconvolution.

45. Add your Far red/Red images to the workflow processor in the first tab.
46. Choose STAR RED CONF image within the file (see Figure 4C).
47. Enter the image and microscope parameters (see Table 2).
48. Enter the deconvolution parameters for the far-red confocal image (Table 3), including the appropriate theoretical or experimentally measured point spread function (PSF), and average value of the signal to noise ratio (see Troubleshooting 1).

**Table 3:** The deconvolution parameters used in this protocol, the settings with an asterisk must be changed based on your images

Setting	Confocal	STED
Algorithm	Classic MLE	Classic MLE
PSF mode	Theoretical	Theoretical
Max. Iterations	40	40
Iteration Mode	Optimized	Optimized
Quality change thresh. (%)	0.05	0.05
Signal to Noise Ratio∗	11.111111	5.555556
Anisotropy mode	Off	Off
Anisotropy	N/A	N/A
Acuity mode	Off	Off
Acuity	N/A	N/A
Background mode	Lowest Value	Lowest Value
Background estimation radius	0.7	0.7
Relative background	0.0	0.0
Bleaching correction	Off	Off
Brick mode	Auto	Auto
PSFs per brick mode	Off	Off
PSFs per brick, manual mode	1	1
Array detector reduction mode	Auto	Auto
Timeout (s)	10000	10000

***Note:*** A theoretical PSF was used for the deconvolution performed in this protocol and provided satisfactory results. Theoretical PSFs are generated from the microscope and acquisition parameters entered in Huygens and are suitable when these parameters are accurately specified. However, they do not capture all setup-specific optical imperfections that may be present in the experimental system. An experimentally measured PSF, obtained using sub-resolution fluorescent beads under acquisition conditions matching those used for the samples, can account for system-specific aberrations and may further improve deconvolution accuracy. When implementing this workflow on a different microscope or with a different objective, either approach can be used. An experimental PSF may be advantageous when available, whereas a theoretical PSF remains an appropriate option provided that all microscope parameters required for PSF generation are entered accurately.

49. Set the save location and the save format as a.ics file.
50. Click “Send and close”.
51. Repeat steps 46–50 for the other channels in your Far Red/Red images changing the parameters.
52. Add your green images to the workflow processor.
53. Repeat steps 46–50 for the STAR GREEN STED images.
54. Click “Run”.
55. Once the deconvolution is done, open the 5 files which are associated with a single image.
56. Open the “Join” function.
57. Merge your files 5 in the order that follows.
  a. Green STED.
  b. Far Red Confocal.
  c. Far Red STED.
  d. Alexa 594 Confocal.
  e. Alexa 594 STED.

***Note:*** The plug-in is pre-programmed to use this order, thus we recommend it to streamline later analysis. There is however a method to alter this order within the plug-in if needed.

***Note:*** Make sure all your images have the same channel order.

***Note:*** It is possible to merge larger batches of files using Fiji macros.

58. Save your images as a.ics or.tif file.

### Measurement of astro-synapse distance

**Timing: 4–6 h (approx. 20 astrocytes)**

This section describes how to use the custom-made Fiji plug-in which we use to analyze the confocal/STED images taken. The plug-in detects the maxima found within the vGlut/Homer STED channel and removes any false positives by checking the presence of maxima’s intensity within the confocal channel as well. It then measures the distance between the Homer and vGlut dots, which if close enough, can be considered a synapse. Finally, the distance between the middle of the synapse and the edge of the astrocyte can be measured (see Figure 5A).

59. Open Fiji/Image J.^5^
60. Install the 3D Image Suite, GDSC, HDF5 and Bio-Formats update sites.

***Note:*** This protocol was successfully performed using Fiji/ImageJ 2.16.0/1.54p, Java 8 (1.8.0_322), on 64-bit Windows 10. The 3D Image Suite,^6^ GDSC, HDF5, and Bio-Formats update sites were enabled. The installed environment included MCIB3D 4.1.7b, Bio-Formats 8.0.0, GDSC ImageJ plugins 2.0-SNAPSHOT, and JHDF5 19.04.1.

61. Install the Vglut_Homer_Astro3D-v1.0.0.jar plugin.
  a. Access the archived Vglut_Homer_Astro3D v1.0.0 software release at https://doi.org/10.5281/zenodo.21903313. The corresponding GitHub repository is available at https://github.com/mainagarnero/Vglut_Homer_Astro3D_archive.
  b. Download Vglut_Homer_Astro3D-v1.0.0.jar from the plugin/directory.
  c. Copy Vglut_Homer_Astro3D-v1.0.0.jar into the Fiji plugins directory.
  d. Restart Fiji.
62. Once installed, a CIRB folder in the Plugins tab of Image J/Fiji is created.
63. Open the plug-in.
64. Choose the folder containing the merged images.

***Note:*** Ensure that the selected image folder remains accessible throughout processing. If an “Unexpected Network Error” occurs, see troubleshooting 8.

65. Indicate the order in which you merged your images in the first tab called “Channels” (see Figure 5B).

***Note:*** If you altered the order from step 57, indicate it in this tab.

***Note:*** Any parameters you alter in this window will be applied to all the compatible images within the chosen folder, so make sure all your files are consistently ordered, named and formatted. If the plug-in displays an “IllegalArgumentException” or “FileNotFoundException” see troubleshooting 7.

***Note:*** Click between the tabs to change other settings. DO NOT click on “OK” until you are ready to launch the plug-in.

66. In the second “Vglut” tab (see Figure 5B).
  a. Choose the “Confocal channel reference intensity”.
    i. The threshold at which detected STED dots will be included when compared to the thresholded confocal image.
      ***Note:*** For this dataset, a value of 5000 was used. Optimal plug-in parameters depend on staining intensity, background, acquisition settings, and image quality. Therefore, this parameter should be optimized on representative images before batch processing (see troubleshooting 6). Once optimized, the selected value should be kept constant across comparable images and experimental groups.
  b. Set the Radius XY (pixel) and Radius Z (pixel).
    i. The size of the maxima which will be included in the final dot count.
      ***Note:*** For this dataset, Radius XY = 3 pixels and Radius Z = 3 pixels were used. Radius values should be selected according to the expected dimensions of the labeled puncta and the voxel dimensions of the acquired images. If the xy pixel size, z-step, or achieved lateral or axial STED resolution is modified, Radius XY and Radius Z should be re-evaluated to maintain an appropriate physical detection neighborhood.
  c. Set the “Noise”.
    i. The threshold at which maxima will be detected within the STED channel.
      ***Note:*** For this dataset, a value of 1500 was used. Optimal plug-in parameters depend on staining intensity, background, acquisition settings, and image quality. Therefore, the parameter should be optimized on representative images before batch processing (see troubleshooting 6). Once optimized, the selected value should be kept constant across comparable images and experimental groups.
67. Repeat steps 66a-66c for the “Homer” tab (see Figure 5C).

***Note:*** vGlut and Homer images may differ in signal intensity, background, labeling density, and punctum morphology. Parameters should therefore be optimized independently for each marker using representative images, as described in troubleshooting 6.

68. In the “Synapses” tab (see Figure 5D).
  a. Set the “Maximum distance between 2 dots (micron)”.
    i. The furthest the maxima of intensity of the two dots can be separated in 3D while still being considered a synapse. For this dataset, a maximum distance of 0.4 μm value was used.
  b. Set the “Maximum distance to astrocyte (micron)”.
    i. The maximum distance between the center of an identified putative synapse and the nearest edge of the segmented astrocyte for the synapse has to be retained for subsequent astrocyte-synapse distance analysis. For this dataset, a maximum distance of 1.5 μm value was used.
      ***Note:*** A relatively large maximum distance can be selected during plug-in processing to estimate the total number of detected synapses. A more restrictive distance cutoff can then be applied during subsequent analysis of the exported data.
  c. Select the channels requiring “Histogram matching bleach correction”.
    i. Applies bleach correction to any channels experiencing significant photobleaching.
      ***Note:*** Select the photobleaching parameters only if necessary as it will increase the processing time significantly. Prioritize avoiding photobleaching altogether during imaging.
      ***Note:*** For large batches of images, unless all images experienced bleaching at similar levels and within the same channel you should not select these parameters. Run photobleached images through the plug-in independently to not apply unnecessary correction to other images.
69. Click on “OK”.
70. Choose the Thresholding method.

***Note:*** Avoid using the “Default” parameters, the “Triangle” and “Otsu” methods are preferred depending on the signal to noise ratio of your images. “Triangle” is particularly suitable when the intensity histogram is dominated by a large background population with a tail of brighter foreground pixels. “Otsu” may perform better when background and labeled structures form more clearly separable intensity populations. In our experience, “Otsu” can produce a more restrictive segmentation, which may reduce background but can also exclude dim fine astrocytic processes. The selected thresholding method should therefore be visually validated on representative images and then kept identical across images and experimental groups acquired under comparable conditions.

71. Click on “OK” not on “Apply”.

***Note:*** The console will display information about the images being processed (see Figure 5E and “expected outcomes” section).

**Figure 5:**
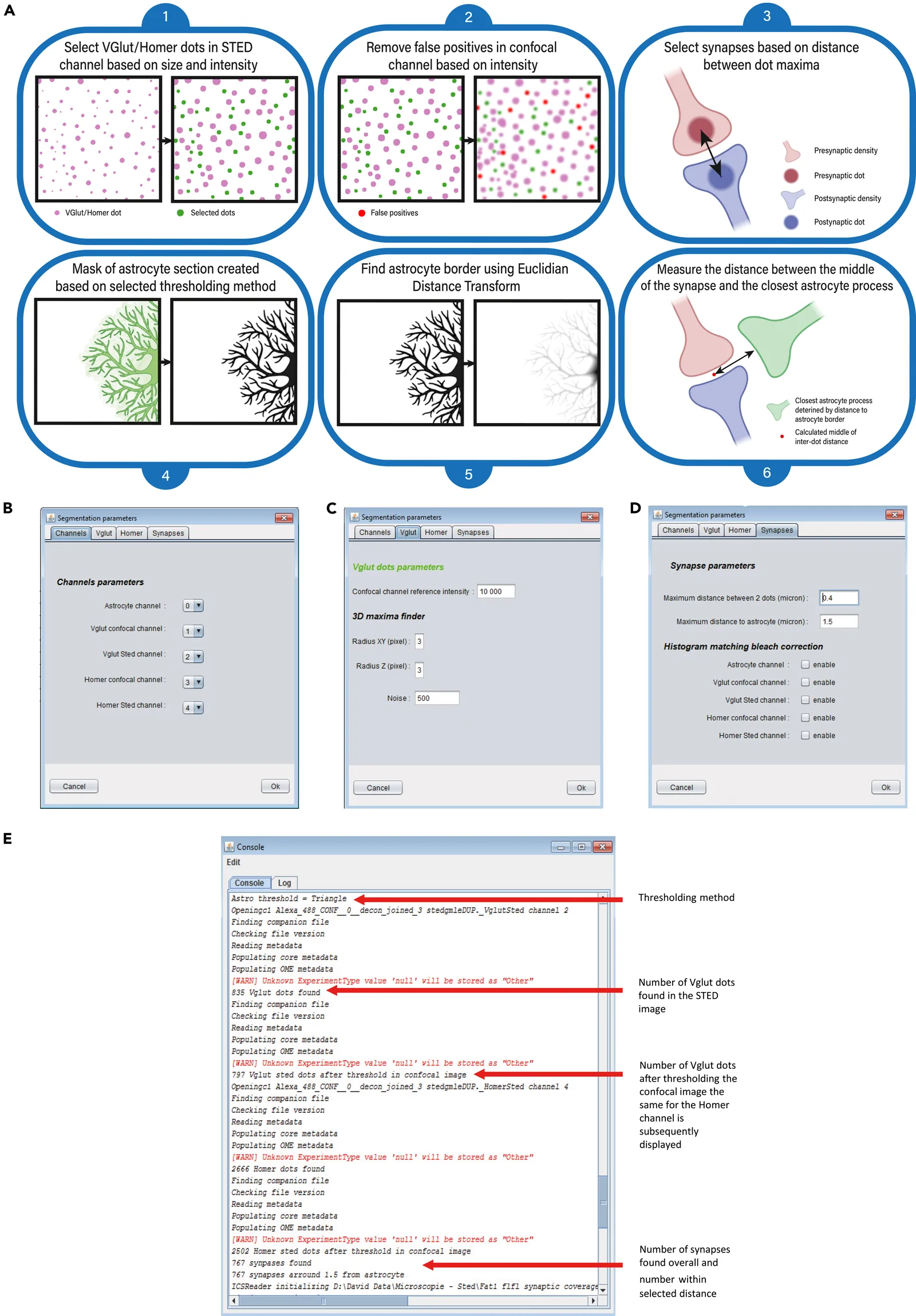
Plug-in interface (A) Overview of the analysis carried out by the custom Fiji plug-in. (B) Channel selection window tab which chooses the order in which the channels have been merged. (C) Vglut dot parameters tab. (D) Synapses parameters tab. (E) Console window. Displays some important information which can be used to ascertain if the plug-in parameters are adapted to the images.

## Expected outcomes

As the plug-in is running, the console will display some key information about the number of maxima in each image and the number of synapses found at the end (see Figure 5E). This is not necessary to record for the final analysis, but is useful to determine which parameters are ideal for your specific images (see Troubleshooting 6). A “Results” folder will be generated in the chosen folder. Within, each image will have three associated files. Two known as “VglutSted_StedinConf” and “HomerSted_StedinConf” will display the detected Vglut and Homer maxima following thresholding in the confocal image. The third, known as “Objects” will display the detected synapses. It is important to know that the plug-in omits the first and last slide since the information above and below the Z-stack is lacking and could create artefacts from out of focus signal. Furthermore, dots are identified at their maxima. Thus, as images are stacks, the dots may be counted as such on one slide while still appearing on another. Finally, the synapse will be tagged as the middle between two dots (that are within the desired distance). The synapse may therefore be in a different slide than the dots that make it up if it is above or below it on the Z axis.

The data itself is kept in an excel file called “Synapses”, once again automatically generated within the “Results” folder (see Figure 6A). It contains the following information: the image name, the volume of the thresholded section of astrocyte, the number of synapses, the distance between the Vglut and Homer dots, the distance between the synapse and the nearest astrocyte process and finally the intensity of the two dots. The data for different images run from the same folder is presented one after the other in the same columns. Thus, the data will need to be extracted and organized to properly analyze it. This can be done manually or through the use of macros.

**Figure 6:**
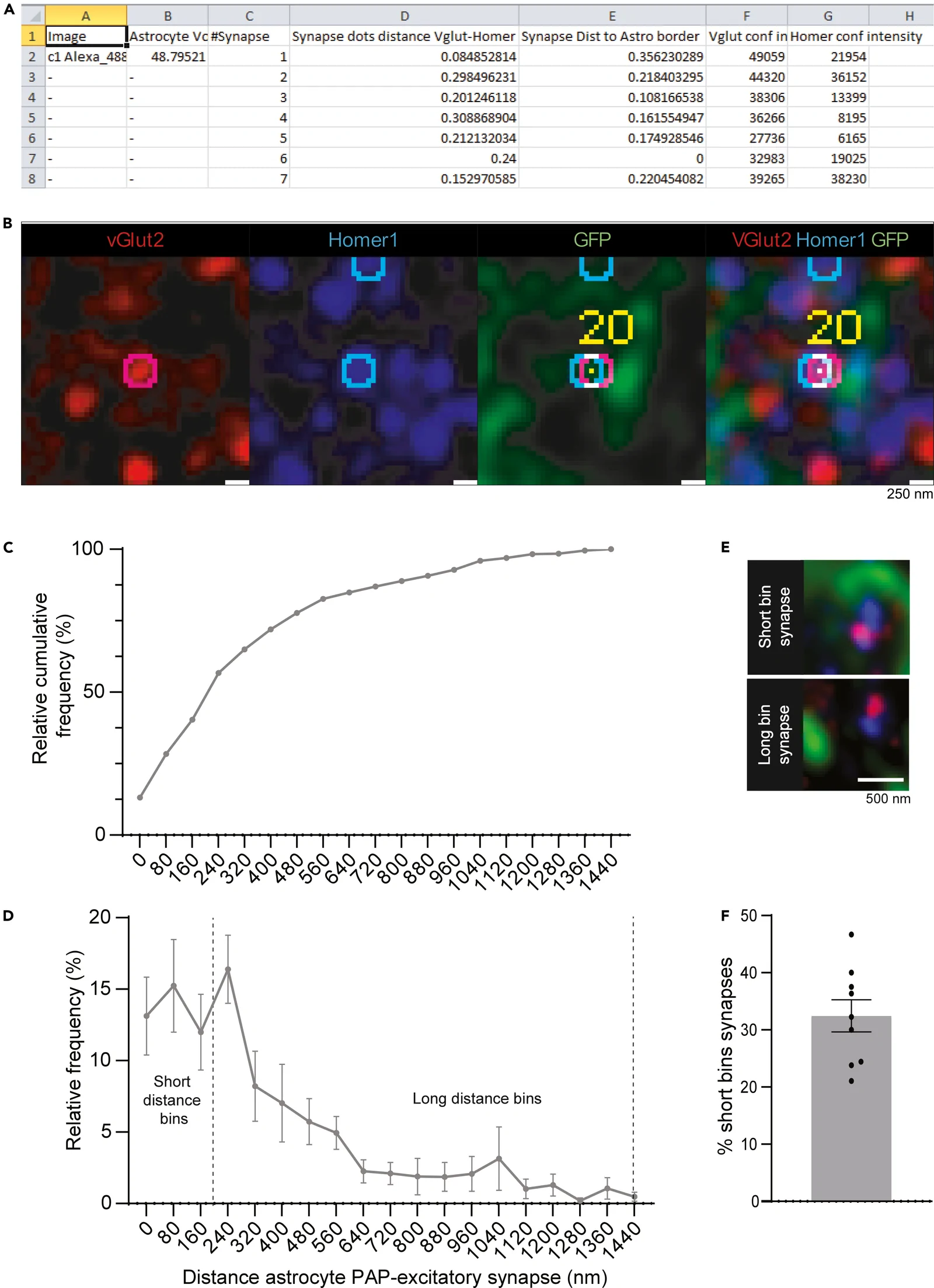
Expected outcomes (A) Data that is generated from the plug-in found within the “Synapses” file. (B) Images generated by the plug-in showing the detected dots in Vglut and Homer. The resulting synapse found between the two detected dots. Finally, an overlay of all these channels. Scale bar: 250 nm. (C) Dot plot of a cumulative frequency analysis obtained using this protocol. (D) Dot plot of a frequency analysis obtained using this analysis pipeline. Data are represented as mean ± SEM. (E) Example of the synapses and their proximity to astrocyte processes. Scale bar: 500 nm. (F) Further analysis done on specific distance bins. Data are represented as mean ± SEM.

By splitting and merging the three generated image files you can properly check the overlap between the dots in the image, whether or not they are picked up by the plug-in and if they are considered as a synapse (see Figure 6B).

For ROIs of 25 × 25 × 1 μm (625 μm^3^), the segmented astrocytic volume typically ranges from approximately 30 to 120 μm^3^, depending on the position of the ROI within the astrocytic arborization. Within the same ROIs, we typically detect approximately 5–40 putative synapses located within 1.5 μm of the segmented astrocyte (Figure 4D). The number of detected synapses can vary according to the brain region and the synaptic population examined (e.g., vGlut1-or vGlut2-positive excitatory synapses or inhibitory synapses).

Using the frequency and cumulative analyses, with bins set to the resolution of your microscope, you can generate figures like this which will nicely show the difference between your two distributions. The relative cumulative frequency corresponds to the result you obtain when applying a Kolmogorov–Smirnov test to the pooled raw data (see Figure 6C). The test does not account for the number of independent replicates per condition, as all observations are combined into a single cumulative distribution. Consequently, variability between replicates is not represented, and no error bars can be derived from this analysis. The dot plot from the frequency distribution allows you to see if there is more of a difference at shorter bins or longer bins (see Figure 6D). Further analysis can be done on specific distance bins for statistical analysis (see Figure 6F).

## Quantification and statistical analysis

**Timing: 1 h 30 min**

The data is found within the “Synapses” file of the automatically generated “Results” folder. The data has a hierarchical structure: multiple synapses are measured within each ROI, multiple ROIs are acquired from each astrocyte, and multiple astrocytes are analyzed from each animal. ROIs therefore represent spatial subsamples within astrocytes, astrocytes represent cellular sampling units, and animals represent independent biological replicates.

For visualization of the overall astrocyte-synapse distance distribution, all individual synapse-to-astrocyte distances from each experimental condition can be pooled and compared using a Kolmogorov–Smirnov test. For subsequent inferential comparisons between experimental groups, individual astrocyte-level measurements can be retained using an appropriate hierarchical or mixed-effects model in which astrocytes are nested within animals. Alternatively, astrocyte-level measurements can be averaged within each animal to obtain one value per biological replicate.

1. Open the automatically generated “Results” folder.
2. Open the “Synapses” excel file (Figure 6A).

***Note:*** Inspect the distance data before proceeding with the statistical analysis. Some images may occasionally produce an unusually high number of zero values or repeated identical distance values. If this occurs, do not include these data in the analysis until the affected images have been checked and reprocessed as described in troubleshooting 2.

***Note:*** If very few putative synapses are detected in the output, verify whether this reflects low signal or suboptimal detection parameters before proceeding with the analysis (see troubleshooting 3).

3. Copy the raw data into your statistical analysis software of choice, such as GraphPad Prism.
4. For visualization of the overall astrocyte-synapse distance distribution, combine the individual synapse-to-astrocyte distances from each experimental condition into a single column.
5. Perform a two-sample Kolmogorov-Smirnov test in GraphPad Prism to compare the overall cumulative distance distributions between conditions.

***Note:*** The Kolmogorov–Smirnov test quantifies the maximum absolute difference between the empirical cumulative distributions of two samples, with significance determined by both this deviation and sample size. Consequently, a statistically significant result can arise from a pronounced local difference between distributions, even if they are otherwise largely similar.

6. Combine the measurements from the three ROIs acquired from each astrocyte to obtain an astrocyte-level dataset.

***Note:*** Individual astrocytes represent cellular sampling units. Multiple astrocytes originating from the same animal should not be considered fully independent biological replicates, as this could constitute pseudoreplication if the animal-level structure is not incorporated into the statistical analysis. Report both the number of astrocytes (n) and the number of animals (N).

7. Run frequency and cumulative frequency analyses for each astrocyte using a bin size appropriate for the spatial resolution of the STED images. In our setup, a bin size of 80 nm is used.
8. Generate a dot plot from the frequency analysis.

***Note:*** Generating a dot plot from this data will allow you to see if there are differences within specific ranges (see Figure 6D). A good way to determine these ranges is to see where the curves intersect.

9. For analysis of a specific distance range, calculate the proportion of detected putative synapses falling within that range for each astrocyte.
10. Display the individual astrocyte-level values to visualize cellular variability within and between animals.
11. Perform inferential comparisons between experimental groups while accounting for the nesting of astrocytes within animals.
  a. One approach is to average the astrocyte-level values from each animal and perform the statistical comparison using one value per animal.
  b. Alternatively, retain the individual astrocyte-level measurements and use an appropriate hierarchical or mixed-effects model in which astrocytes are nested within animals.
12. When animal-level summary values are compared between two independent experimental groups, inspect the distribution of the biological replicate values and select an appropriate statistical test. An unpaired *t* test may be used after checking the normality of the data. Otherwise, a Mann–Whitney test may be used.

## Limitations

This protocol presents a few limitations. Firstly, regardless of the protocol used (transgenic line or viral injection), we are assessing only a sample of the overall astrocytic population. The GFAP promoter used in this protocol only stains a subtype of astrocytes. The conclusions drawn from this experiment will thus only apply to this specific subpopulation.

An additional limitation concerns the measurement of distances along the z-axis. In this protocol, images are acquired using 3D-STED rather than 2D-STED, providing improved resolution in both the lateral and axial dimensions. Nevertheless, axial resolution remains lower than lateral resolution and can be affected by factors such as refractive-index mismatch, optical aberrations, labeling quality, and the z-step used for acquisition. Consequently, the uncertainty associated with the z-component of a three-dimensional distance measurement is greater than that associated with the x y components, particularly for structures separated by distances approaching the axial resolution limit. Astrocyte-synapse distances should therefore be interpreted with this limitation in mind, and acquisition parameters, including the 3D-STED mode and z-step, should be kept consistent across experimental groups.

Furthermore, the results produced by the plug-in are heavily dependent on the quality of your images and subsequent pre- and post-processing. Strong background and photobleaching will affect the results, especially if this is inconsistent between images.

Finally, the thresholding methods and parameters used for the plug-in will yield very different outcomes even using the same batch of images. Selecting the “Triangle” thresholding method over the “Otsu” for example will mean that more of the astrocyte will be taken into account, thus increasing the likelihood of finding synapses closer to the astrocyte. In terms of the parameters, increasing the threshold in the confocal channel will mean fewer dots will be taken into account, decreasing the number of synapses found, which will obviously affect the final data. Using the mean parameters for the plug-in will reduce this variability and allow for consistent processing/analysis of all your images. However, the choice of parameters will inevitably rely on some subjective assessment of the difference between the number of dots detected in the confocal and STED channels.

## Troubleshooting

### Problem 1

Determining Signal to Noise Ratio during deconvolution (relevant to “deconvolution” section – step 48).

**Figure 7:**
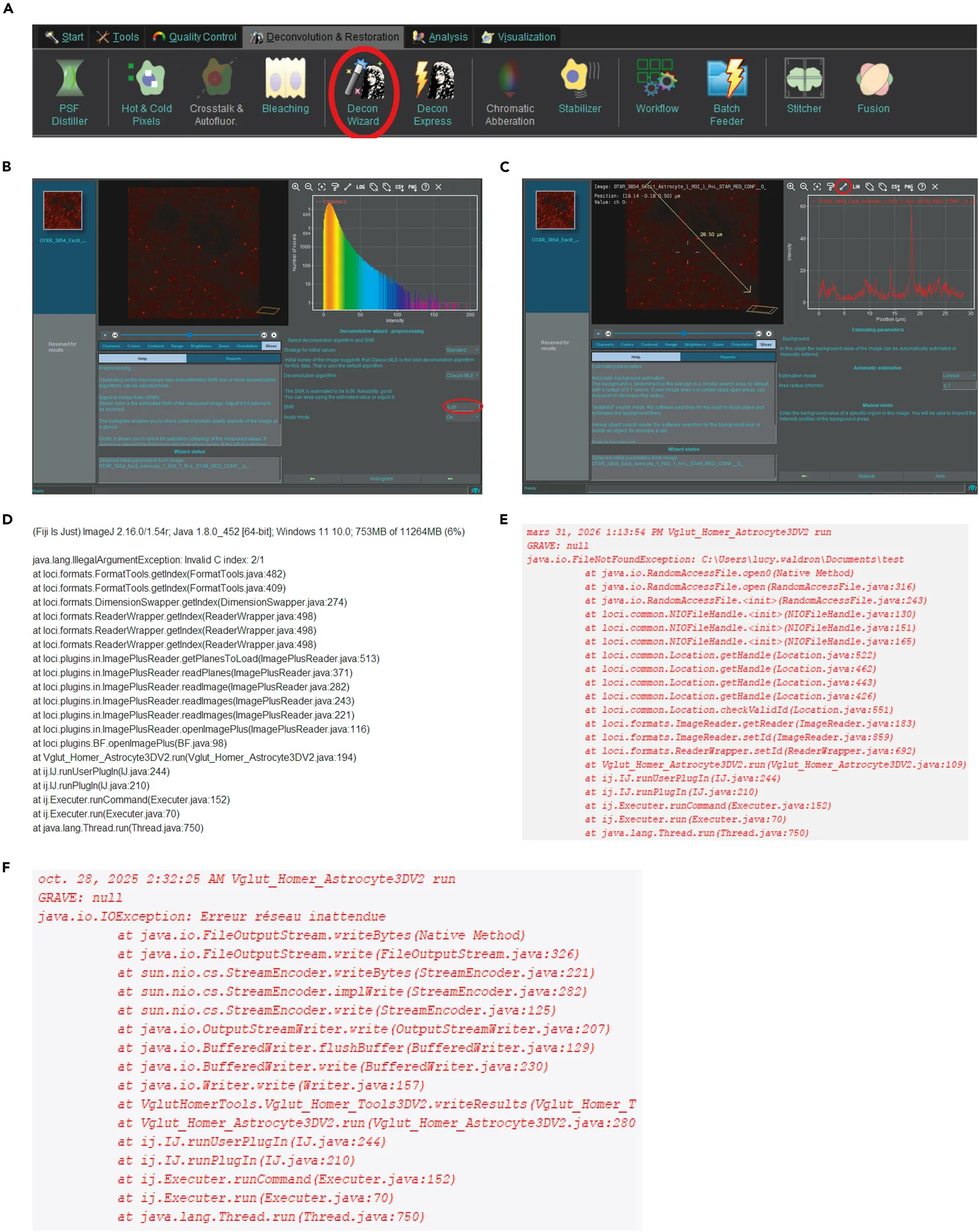
Troubleshooting (A) Huygens Pro Toolbar with the deconvolution wizard button circled. (B) Automatically determined signal to noise ratio. (C) Manual determined signal to noise ratio using the drawing tool and the histogram. (D) Error message following plug-in trying to use an image with the wrong number of channels. (E) Error message displayed following plug-in trying to process an incompatible format. (F) Error message displayed following a loss of access to the image folder.

### Potential solution

It is very important that you find an average value of the signal to noise ratio (SNR) for your images.

• Choose 5–10 images which represent a wide variety of your image population (across conditions, acquisition days/times, animals, slices etc.).
• Load these up into Huygens Pro.
• Click on the deconvolution wizard (see Figure 7A).
• The first sections are not important but input the classic MLE.
• In the tab in figure x, Huygens will give you an estimation of the SNR.
• You can check it yourself in the next tab (see Figures 7B and 7C).
  ◦ Draw a line over your image across points of interest as well as the background.
  ◦ Here you will be able to estimate your SNR by ratioing the peak of your signal with the background.
• Do this for your remaining images.
• Take an average of these SNRs.
• You will be able to use this value once you process all your images.

### Problem 2

When looking at the distance data, some of the images will have repeating distance values and many zeros (relevant to “quantification and statistical analysis” section – step 73).

### Potential solution

• The plug-in sometimes has issues running large numbers of images at once. Run these images independently back through the plug-in, as described in “measurement of astro-synapse distance” section – step 70, with all the same settings.
• It can also be that your thresholding method is not adapted to your images, and thus too much of the background is being considered as part of the synapse. Change the thresholding method and re-run these images through the plug-in, as described in “measurement of astro-synapse distance” section – step 70.
• If this does not help, then return to the “deconvolution” section – step 48, and change the deconvolution parameters to remove more of the background.

### Problem 3

Detecting very few synapses (relevant to “quantification and statistical analysis” section – step 73).

### Potential solution

• Reduce dot detection threshold, as described in the “measurement of astro-synapse distance” section. This increases the likelihood of finding synapses.
• Increase the laser power/alter the microscope parameters (such as dwell time) to increase the signal during imaging (see “setting up the STED microscope” and “imaging astrocytes with STED” sections).

### Problem 4

Trouble finding isolated astrocytes (relevant to “before you begin” section, and “imaging astrocytes with STED” section – step 18).

### Potential solution

There are a few solutions:

• It is not necessary to find a totally isolated astrocyte (although that is ideal), as long as your target astrocyte has enough isolated processes to accommodate 3 distinct ROIs then it can be used for imaging.
• If the targeted astrocyte is much more intense than those around it then it can still be used. Proper deconvolution and thresholding will help isolate it from those around.

### Problem 5

Photobleaching (relevant to “setting up the STED microscope” section – step 9, and “imaging astrocytes with STED” section).

### Potential solution

There are several methods to reduce photobleaching:

• Reducing the laser power.
• Reducing the dwell time and increasing the number of line accumulations.
• Reducing the stack size.

### Problem 6

Choosing parameters for the plug-in (relevant to “measurement of astro-synapse distance” section – steps 66–67).

### Potential solution

The same parameters will produce very different results based on each image so it is imperative to get find parameters which will work for the majority of your images. To do this:

• Take 5–10 images, which are a good representation of your images overall (different conditions, different labelling experiments…).
• Run these files through the plug-in with different parameters and take note of the number of dots, which are detected before and after thresholding in both the Vglut and Homer channels.
• Visually inspect the detected puncta together with the original STED and confocal images in the generated StedinConf.tif files for both Vglut and Homer. The aim is to identify parameters that retain genuine puncta while minimizing false-positive detections and preventing excessive exclusion of dim but valid puncta.
  ***Note:*** A difference between the number of puncta initially detected in the STED channel and the number retained after confocal validation is expected. For example, if 1,300 puncta are detected in the STED image and 800 are retained after confocal validation, the excluded puncta may represent false-positive STED detections, an overly stringent confocal reference-intensity threshold, or a combination of both. The objective is therefore not to obtain identical STED and confocal puncta counts, but to achieve reliable detection based on visual validation.
  ○ If for example you feel that too many false positive dots are being picked up in the STED image, then you can increase the value of the threshold “Noise” parameter. If genuine but relatively dim puncta are not detected, decrease the “Noise” parameter.
  ○ On the other hand, if you feel that following thresholding in the confocal channel, too many dots are being excluded then you can reduce the threshold in the “Confocal channel reference intensity” parameter (to around 500–2000 for example).
  ○ The parameters which get you as close to those ideal conditions across the sample of images should be the ones you use for all your files.

### Problem 7

Plug-in displays error message in separate window with “IllegalArgumentException” or “FileNotFoundException” (Figures 7D and 7E) (relevant to “measurement of astro-synapse distance” section – steps 64–65).

### Potential solution

• Make sure all your images have a compatible file type and the correct number of channels within the same batch of files.
  ○ Even if all the files can be read the plug-in sometimes has trouble switching between compatible file types within the same run.

### Problem 8

Error displayed in console “java.io.IOException: Unexpected Network Error» (Figure 7F) (relevant to “measurement of astro-synapse distance” section - steps 64–65).

### Potential solution

Make sure the plug-in has access to the folder containing your images at all times throughout the run.

## Resource availability

### Lead contact

Requests for additional information, resources, or reagents can be made to the lead contact, Nathalie Rouach (nathalie.rouach@college-de-france.fr).

### Technical contact

Questions regarding the execution of the protocol can be posed to the technical contacts, Lucy Waldron (lucy.waldron@college-de-france.fr) and Maïna Garnero (maina.garnero@college-de-france.fr).

### Materials availability

This study did not generate new unique reagents.

### Data and code availability

• The Vglut_Homer_Astro3D Fiji/ImageJ plug-in used in this protocol is available as an archived software release (v.1.0.0) on Zenodo: https://doi.org/10.5281/zenodo.21903313. The corresponding source-code repository is available at https://github.com/mainagarnero/Vglut_Homer_Astro3D_archive.
• The archived release contains the Java source code and the compiled Vglut_Homer_Astro3D-v1.0.0.jar used for the analyses.
• The plug-in was successfully run using Fiji/ImageJ 2.16.0/1.54p, Java 8 (1.8.0_322), on 64-bit Windows 10. The 3D Image Suite, GDSC, HDF5, and Bio-Formats update sites were enabled. The installed environment included MCIB3D 4.1.7b, Bio-Formats 8.0.0, GDSC ImageJ plug-ins 2.0-SNAPSHOT, and JHDF5 19.04.1.
• This study did not generate datasets requiring deposition in a public repository.

## Acknowledgments

The custom Fiji plug-in was designed and written by Phillippe Mailly of the ORION imaging facility of the Center for Interdisciplinary Research in Biology, Collège de France. The graphical abstract and figures adapt objects from BioRender.com. This work was supported by grants from Ecole Normale Supérieure Paris-Saclay and the 10.13039/501100002915Fondation pour la Recherche Médicale (FRM, FDT202604051526) to M.G. (PhD grants), the Chaire D’Excellence Biologie-Santé (SensoPAP, ANR-24-CHBS-0007), ANR (AstroXcite, ANR-22-CE16-0027) and the Major Research Program of PSL Research University “PSL-Neuro,” launched by PSL Research University and implemented by ANR (ANR-10-IDEX-0001) to N.R.

## Author contributions

Conceptualization, N.R., D.M., M.G., and L.W.; data collection, M.G.; data analysis and interpretation, M.G. and L.W.; writing of the original draft, M.G. and L.W.; editing of the original draft, M.G., L.W., D.M., and N.R.; revision of the manuscript, M.G. and N.R. The final version was reviewed and approved by all authors.

## Declaration of interests

The authors declare no competing interests.

## Declaration of generative AI and AI-assisted technologies in the manuscript preparation process

During the preparation of this work, the authors used ChatGPT (OpenAI) in order to assist with language editing, improving clarity and readability, and refining the organization of the protocol. After using this tool/service, the authors reviewed and edited the content as needed and take full responsibility for the content of the publication.
